# Management of metastatic colorectal cancer in patients ≥70 years - a single center experience

**DOI:** 10.3389/fonc.2023.1222951

**Published:** 2023-07-25

**Authors:** Florian Huemer, Celine Dunkl, Gabriel Rinnerthaler, Konstantin Schlick, Ronald Heregger, Klaus Emmanuel, Daniel Neureiter, Eckhard Klieser, Michael Deutschmann, Falk Roeder, Richard Greil, Lukas Weiss

**Affiliations:** ^1^ Department of Internal Medicine III with Haematology, Medical Oncology, Haemostaseology, Infectiology and Rheumatology, Oncologic Center, Salzburg Cancer Research Institute - Center for Clinical Cancer and Immunology Trials (SCRI-CCCIT), Paracelsus Medical University Salzburg, Salzburg, Austria; ^2^ Cancer Cluster Salzburg, Salzburg, Austria; ^3^ Department of Surgery, Paracelsus Medical University Salzburg, Salzburg, Austria; ^4^ Institute of Pathology, Paracelsus Medical University Salzburg, Salzburg, Austria; ^5^ Department of Radiology, Paracelsus Medical University Salzburg, Salzburg, Austria; ^6^ Department of Radiation Oncology, Paracelsus Medical University Salzburg, Salzburg, Austria; ^7^ Tumor Registry of the Province of Salzburg, Salzburg, Austria

**Keywords:** elderly, age, ECOG performance score, colorectal cancer, sidedness, local ablative treatment, metastasectomy

## Abstract

**Background:**

Age-standardized mortality rates for metastatic colorectal cancer (mCRC) are highest among elderly patients. In current clinical guidelines, treatment recommendations for this patient population are based on a limited number of clinical trials.

**Patients and methods:**

In this monocentric, retrospective analysis we characterized patients aged ≥70 years undergoing systemic therapy for mCRC and overall survival (OS) was investigated.

**Results:**

We included 117 unselected, consecutive mCRC patients aged ≥70 years undergoing systemic therapy for mCRC between February 2009 and July 2022. Median OS was 25.6 months (95% CI: 21.8-29.4). The median age was 78 years (range: 70-90) and 21%, 48%, 26% and 5% had an ECOG performance score of 0, 1, 2, and 3, respectively. The median number of systemic therapy lines was 2 (range: 1-5). The choice of first-line chemotherapy backbone (doublet/triplet versus mono) did not impact OS (HR: 0.83, p=0.50) or the probability of receiving subsequent therapy (p=0.697). Metastasectomy and/or local ablative treatment in the liver, lung, peritoneum and/or other organs were applied in 26 patients (22%) with curative intent. First-line anti-EGFR-based therapy showed a trend towards longer OS compared to anti-VEGF-based therapy or chemotherapy alone in left-sided mCRC (anti-EGFR: 39.3 months versus anti-VEGF: 27.3 months versus chemotherapy alone: 13.8 months, p=0.105). In multivariable analysis, metastasectomy and/or local ablative treatment with curative intent (yes versus no, HR: 0.22, p<0.001), the ECOG performance score (2 versus 0, HR: 3.07, p=0.007; 3 versus 0, HR: 3.66, p=0.053) and the presence of liver metastases (yes versus no, HR: 1.79, p=0.049) were independently associated with OS.

**Conclusions:**

Our findings corroborate front-line monochemotherapy in combination with targeted therapy as the treatment of choice for elderly mCRC patients with palliative treatment intent. Metastasectomy and/or local ablative treatment with curative intent are feasible and may improve OS in selected elderly mCRC patients.

## Introduction

Colorectal cancer (CRC) is the second most common cause of cancer-related death worldwide (1). The incidence rate of CRC considerably increases with age and age-standardized CRC mortality rates are highest among elderly patients (2, 3). Elderly metastatic CRC (mCRC) patients (≥70 years) are underrepresented in clinical trials and one out of four elderly mCRC patients does not receive chemotherapy-based palliative systemic therapy due to comorbidities, chronological age or poor performance status (4).

Therapeutic decision making and treatment recommendations by the European Society of Medical Oncology (ESMO) (5) and National Comprehensive Cancer Network (NCCN) (6) for elderly mCRC patients are mainly based on a limited number of clinical trials focusing on the elderly mCRC population (7–9). Fluorouracil-based monochemotherapy in combination with anti-VEGF-based therapy irrespective of sidedness (7, 9) or in combination with anti-EGFR-based therapy (8) as well as anti-EGFR monotherapy (10) in patients with RAS wild-type left-sided tumors represent recommended first-line protocols (5). A median overall survival of 14 and 21 months is achieved with the abovementioned first-line protocols among patients ≥75 years (10) and ≥70 years (7), respectively; however, data on the clinical outcome in the elderly mCRC population in the real-world setting are sparse.

While metastasectomy and/or local ablative treatment (+/- perioperative chemotherapy or previous conversion therapy) represent established approaches in eligible patients with oligometastatic CRC (5, 6), there is a paucity of evidence supporting this treatment concept with putative curative intent in the elderly oligometastatic CRC population.

The primary aim of this unicentric retrospective analysis was to evaluate the therapeutic management of mCRC patients ≥70 years of age and clinical outcome in a real-world setting. Furthermore, this analysis aimed at investigating the frequency, feasibility and efficacy of metastasectomy and/or local ablative treatment with putative curative intent in this elderly population.

## Patients and methods

### Patients

This retrospective analysis was approved by the Ethics Committee of the provincial government of Salzburg, Austria (415-E/2343/5-2018). Patients with an age ≥70 years at the time point of histologically confirmed mCRC diagnosis and who received systemic therapy for mCRC at our tertiary cancer center (Department of Internal Medicine III, Paracelsus Medical University Salzburg, Austria) between February 2009 and July 2022 were included in this analysis. All included patients alive at the date of analysis signed an informed consent form. Early access within a named patient program was available for patients who had received regorafenib and/or TAS-102 before the respective approval by the European Medicines Agency (EMA). Data were extracted from medical records, including:

1. patient characteristics: mCRC diagnosis date, age, sex, Eastern Cooperative Oncology Group (ECOG) performance score
2. tumor characteristics: time point of metastases detection (synchronous versus metachronous), sidedness (right versus left), histological grade, metastatic distribution pattern at mCRC diagnosis, predictive tumor-tissue-based biomarkers (KRAS-, NRAS-, BRAF-, microsatellite-/mismatch-repair-status)
3. systemic therapy characteristics: number of systemic therapy lines, first-line chemotherapy backbone (mono- versus doublet or triplet chemotherapy), application of targeted therapy during first-line (no antibody versus anti-VEGF versus anti-EGFR), regorafenib and/or TAS-102 exposure and
4. local ablative treatment with curative intent: metastasectomy, microwave ablation (MWA), radiofrequency ablation (RFA), stereotactic body radiation therapy (SBRT), transarterial chemoembolization (TACE) and involved organ(s): liver, lung, peritoneum, other.

In order to draw a comparison in regard to age distribution and treatment intent between our unicentric elderly mCRC cohort and mCRC patients ≥70 years in the province of Salzburg (Austria), data from the Tumor Registry of the Province of Salzburg from 2013 to 2020 were used.

### Statistical analyses

Baseline characteristics were compared using crosstabulation together with the chi-squared test, in case of categorical data. Continuous data were summarized using medians and ranges and compared between groups with the Mann-Whitney test. Uni- and multivariable analyses were based on Cox proportional hazard models. For multivariable analysis covariable selection, a backward stepwise procedure was performed using the Akaike information criterion (AIC) as selection criterion (11). OS was calculated from the date of mCRC diagnosis until death from any cause. Metastasectomy and/or local ablative treatment (yes versus no) as well as regorafenib and/or TAS-102 exposure were taken into account as time-dependent covariates, respectively. Patients alive at the last contact were censored. IBM SPSS Statistics version 27 (Armonk, NY, US) and the statistical software environment R (version 4.1.2, survival and MASS package) were used for statistical analyses. The complete data set is available from the corresponding author on reasonable request.

## Results

### Baseline characteristics

In this retrospective monocentric analysis, 117 mCRC patients aged ≥70 years, diagnosed between February 2009 and July 2022, and undergoing systemic therapy for mCRC were included. The baseline characteristics are depicted in **Table 1**.

**Table 1:** Baseline characteristics of entire elderly mCRC cohort.

Parameter	N=117 (%)
Age (median) Range	78 70-90
Age category 70-74 75-79 80-84 ≥85	36 (31) 42 (36) 32 (27) 7 (6)
Sex Female Male	48 (41) 69 (59)
ECOG performance score 0 1 2 3 NA	24 (21) 55 (48) 30 (26) 5 (5) 3
Time point of metastases detection Synchronous Metachronous	87 (74) 30 (26)
Sidedness Left Right	76 (65) 41 (35)
Exact primary tumor localization Rectum Sigmoid colon Descending colon Left flexure Transverse colon Right flexure Ascending colon Cecum	32 (27) 35 (30) 5 (4) 4 (3) 6 (5) 3 (3) 15 (13) 17 (15)
Histological grade 1 2 3 NA	8 (8) 67 (66) 26 (26) 16
Involved organs at mCRC diagnosis* Liver Lung Peritoneum	80 (68) 40 (34) 22 (19)
KRAS status Wild-type KRAS G12C mutant Non-KRAS G12C mutant NA	49 (47) 4 (4) 51 (49) 13
NRAS status Wild-type Mutant NA	66 (97) 2 (3) 49
BRAF status Wild-type V600E mutant Non-V600E mutant NA	61 (91) 5 (8) 1 (1) 50
Microsatellite/Mismatch-repair status MMRp/MSS MMRd/MSI NA	48 (92) 4 (8) 65
1L chemotherapy backbone Mono chemotherapy Doublet or triplet chemotherapy NA (anti-PD-1 therapy)	32 (28) 83 (72) 2
1L anti-VEGF or anti-EGFR therapy None Anti-VEGF Anti-EGFR	35 (30) 61 (52) 21 (18)
Regorafenib and/or TAS-102 exposure None Regorafenib only TAS-102 only Regorafenib followed by TAS-102 (or vice versa)	91 (78) 4 (3) 13 (11) 9 (8)
Metastasectomy and/or local ablative treatment with curative intent No Yes	91 (78) 26 (22)

*multiple designations possible.

ECOG, Eastern Cooperative Oncology Group; mCRC,metastatic colorectal cancer; MMRp, mismatch-repair proficient; MMRd, mismatch-repair deficient; MSI, microsatellite instability; MSS, microsatellite stability.

### Patient characteristics

The median age at mCRC diagnosis was 78 (range: 70-90). 21%, 48%, 26% and 5% had an ECOG PS of 0, 1, 2, and 3 with a median age of 75, 78, 78.5, and 82 years at mCRC diagnosis, respectively (p=0.087).

### Tumor characteristics

Eighty-seven patients (74%) were diagnosed with synchronous mCRC. The primary tumor location was left-sided in 76 patients (65%). Liver, lung and peritoneal metastases were detected in 80 (68%), 40 (34%) and 22 (19%) patients at the time point of mCRC diagnosis, respectively.

Among patients with available tumor-tissue-based biomarkers, KRAS-mutations, NRAS-mutations, BRAF V600E-mutations and MSI/MMRd were detected in 53%, 3%, 8% and 8%, respectively.

### Systemic therapy characteristics

In first line, a monochemotherapy backbone was applied in 32 patients (28%), whereas 83 patients (72%) received a doublet or triplet chemotherapy backbone. The likelihood of applying a doublet or triplet chemotherapy backbone declined with increasing age (p<0.001, **Table A.1**) and with a worse ECOG PS (p=0.007, **Table A.1**). Two patients with MSI/MMRd received immune-checkpoint blockade as palliative first-line therapy.

Sixty-one patients (52%) were treated with anti-VEGF-based therapy in first line, whereas anti-EGFR based therapy was applied in 21 patients (18%). The remaining 35 patients (30%) did not receive targeted therapy in first-line. Anti-VEGF-based therapy, anti-EGFR-based therapy or no targeted therapy were documented in 38 (50%), 14 (18%), and 24 (32%) patients with left-sided and in 23 (56%), 7 (17%) and 11 (27%) patients with right-sided primary tumor localization (p=0.812).

The EMA approved third-line therapy options, regorafenib and TAS-102, were applied in 26 patients (22%) during the course of disease (only regorafenib: n=4 (3%), only TAS-102: n=13 (11%), regorafenib followed by TAS-102 or vice versa: n=9 (8%)).

**Figure 1:**
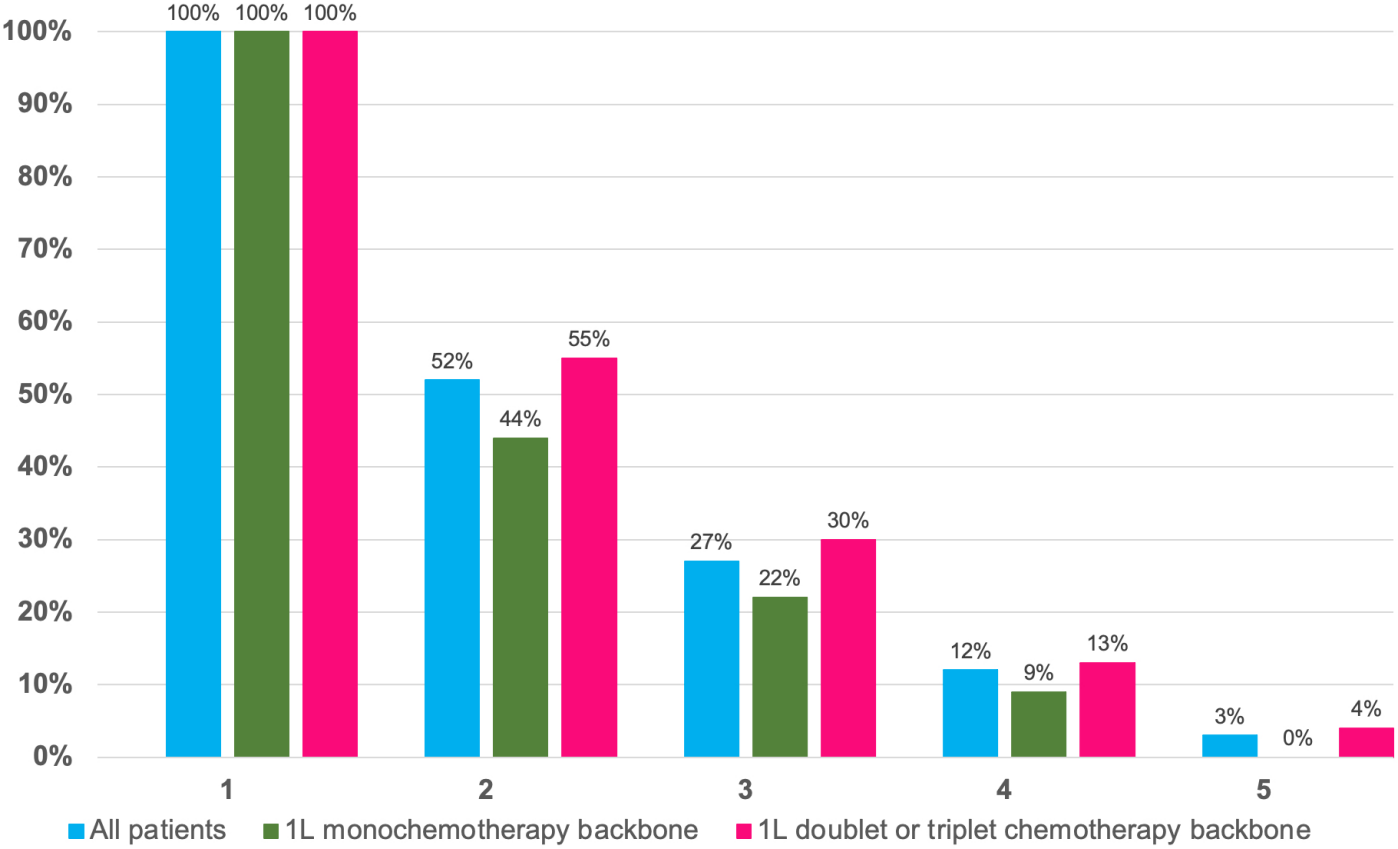
Impact of first-line chemotherapy backbone on number of subsequent therapy lines. Relative number of systemic therapy lines among elderly mCRC patients undergoing first-line therapy with any systemic therapy (blue), a monochemotherapy backbone (green) or a doublet or triplet chemotherapy backbone (red).

The median number of systemic therapy lines in the study population was 2 (range: 1-5) and 52%, 27% 12% and 3% received a second-line, third-line, fourth-line and fifth-line therapy (**Figure 1**). The chemotherapy backbone in first line (mono versus doublet/triplet) did not statistically significantly impact the probability of receiving subsequent therapy (p=0.697, **Figure 1**).

### Metastasectomy and/or local ablative treatment with curative intent

Twenty-six patients (22%) underwent metastasectomy and/or local ablative treatment of metastases in the liver, lung, peritoneum or other organs with curative intent during their course of disease (**Table A.2**):

In twenty-three patients (20%) surgical metastasectomy was performed once, whereas nine (8%) and two patients (2%) underwent metastasectomy twice and three times during their course of disease, respectively. Stereotactic body radiation therapy (SBRT), radiofrequency ablation (RFA) or microwave ablation (MWA), and transarterial chemoembolization (TACE) were applied in six (5%), six (5%) and two (2%) cases, respectively.

Patients undergoing metastasectomy and/or local ablative treatment were more likely to receive a front-line doublet or triplet chemotherapy backbone (89% versus 67%, p=0.035) and showed a trend towards metachronous metastases (38% versus 22%, p=0.090) compared to patients without ablative measures (**Table A.3**).

### Age and treatment intent of elderly mCRC patients in the province of Salzburg

According to the Tumor Registry of the Province of Salzburg (Austria), the following age distribution pattern was found between 2013 and 2020 in the province of Salzburg among mCRC patients ≥70 years: 70-74 years: 32%; 75-79 years: 35%; 80-84 years: 33%, ≥85 years: 0%.

Fifty-nine per cent of the abovementioned patients received palliative systemic therapy and the likelihood decreased with increasing age: 70-74 years: 74%, 75-79 years: 58%; 80-84 years: 44%.

### Overall survival

After a median follow up of 38.4 months (95% CI: 29.3-47.5 months), the median OS in the entire monocentric cohort was 25.6 months (95% CI: 21.8-29.4 months).

#### Univariable analyses

##### Patient-associated factors

A worse ECOG PS at diagnosis was associated with inferior OS (1 versus 0, HR: 1.45, p=0.24; 2 versus 0, HR: 1.58, p=0.22; 3 versus 0, HR: 4.97, p=0.01; **Table 2**). Chronological age at mCRC diagnosis did not impact survival (HR: 1.02, p=0.54; **Table 2**).

**Table 2:** Univariable analysis for overall survival.

Univariable analysis				
Parameter	N	HR	95% CI	p-value
Age (continuous)	117	1.02	0.96-1.08	0.54
Sex Female Male	48 69	1.11	0.69-1.80	0.66
ECOG PS 0 1 2 3	24 55 30 5	1.45 1.58 4.97	0.78-2.69 0.76-3.31 1.37-18.04	0.24 0.22 0.01
Histological grade 1 2 3	8 67 26	0.89 1.35	0.32-2.53 0.45-4.06	0.83 0.59
Sidedness Right-sided Left-sided	41 76	1.10	0.67-1.81	0.71
Liver metastases No Yes	37 80	1.82	1.07-3.09	0.03
Lung metastases No Yes	77 40	0.94	0.57-1.53	0.79
Peritoneal metastases No Yes	95 22	0.52	0.26-1.05	0.07
Time point of metastases detection Metachronous Synchronous	30 87	1.40	0.80-2.46	0.24
KRAS status Wild-type Mutant	49 55	1.06	0.65-1.74	0.80
1L chemotherapy backbone Mono Doublet/triplet	32 83	0.83	0.48-1.43	0.50
Regorafenib and/or TAS-102 exposure^#^ No Yes	50 24	1.14	0.63-2.08	0.67
Number of therapy lines 1 ≥2	56 61	0.40	0.25-0.65	<0.001
Metastasectomy and/or local ablative treatment with curative intent^#^ No Yes	57 25	0.16	0.08-0.33	<0.001

ECOG, Eastern Cooperative Oncology Group.

^#^time-dependent covariate.

##### Tumor-associated factors

**Figure 2:**
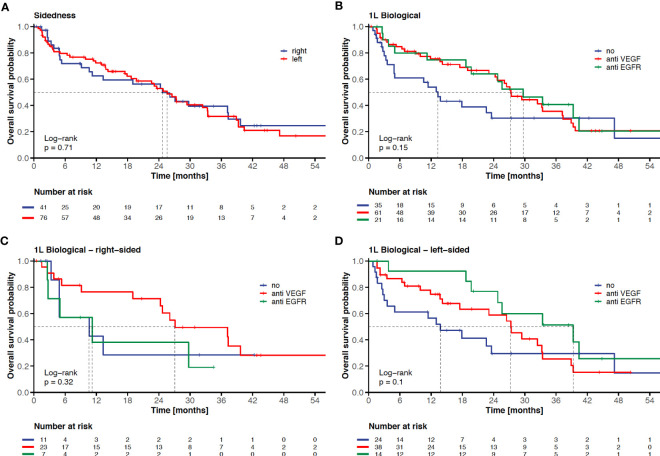
Impact of sidedness and 1L-targeted therapy on clinical outcome in elderly mCRC patients. KM-curves for overall survival according to sidedness (left-sided versus right-sided) **(A)**, according to 1L-targeted therapy (no targeted therapy versus anti-VEGF-based therapy versus anti-EGFR-based therapy **(B)**, according to 1L-targeted therapy in right-sided mCRC **(C)**, and according to 1L-targeted therapy in left-sided mCRC **(D)**. The tick marks on the curves represent censored patients.

Neither sidedness (left-sided versus right-sided, HR: 1.10 p=0.71 log-rank, **Figure 2A**), nor KRAS mutational status (mutant versus wild-type, HR: 1.06 p=0.80; **Table 2**) proved as prognostic factors. The presence of liver metastases at the time point of mCRC diagnosis negatively influenced OS (present versus absent, HR: 1.82, p=0.03; **Table 2**).

##### Systemic therapy

The chemotherapy backbone of front-line therapy did neither affect OS in the entire cohort (doublet or triplet versus monochemotherapy, HR: 0.83, p=0.50; **Table 2**), nor among patients without metastasectomy and/or local ablative treatment (HR: 1.11, p=0.73). The addition of an anti-EGFR or anti-VEGF monoclonal antibody to chemotherapy in first line irrespective of the primary tumor localization resulted in a trend towards longer survival (anti-EGFR: 29.7 months versus anti-VEGF: 27.3 months versus no targeted therapy: 13.3 months, p=0.15 log-rank, **Figure 2B**). The choice of targeted therapy according to sidedness in first-line was associated with a trend towards superior survival with anti-EGFR-based therapy in left-sided disease (anti-EGFR: 39.3 months versus anti-VEGF: 27.3 months versus no targeted therapy: 13.8 months, p=0.105 log-rank; **Figure 2D**), while sidedness proved less predictive in right-sided disease (anti-VEGF: 27.1 months versus anti-EGFR: 11.2 months versus no targeted therapy: 10.6 months, p=0.325 log-rank; **Figure 2C**).

The application of more systemic therapy lines was associated with improved OS (≥2 versus 1, HR: 0.40, p<0.001; **Table 2**). Patients receiving regorafenib and/or TAS-102 during the course of disease did not show a survival benefit (yes versus no, HR: 1.14, p=0.67; **Table 2**) when considered as a time-dependent covariate. Seven patients were treated within clinical trials in first line and three patients in subsequent therapy lines.

##### Ablative therapies

Performing metastasectomy and/or applying local ablative treatment with curative intent statistically significantly improved OS (yes: 47.2 months versus no: 17.9 months, HR: 0.16, p<0.001, **Table 2**). The six-month survival rate was 100% after metastasectomy (liver, lung, peritoneum, other), SBRT (liver, lung), RFA/MWA (liver) and TACE (liver), respectively.

#### Multivariable analysis

Based on a backward stepwise regression the following covariates were selected for multivariable analysis: sidedness (left-sided versus right-sided), liver metastases (present versus absent), ECOG PS (0 versus 1, 0 versus 2, 0 versus 3), regorafenib and/or TAS-102 exposure (yes versus no) and metastasectomy and/or local ablative treatment (yes versus no).

**Figure 3:**
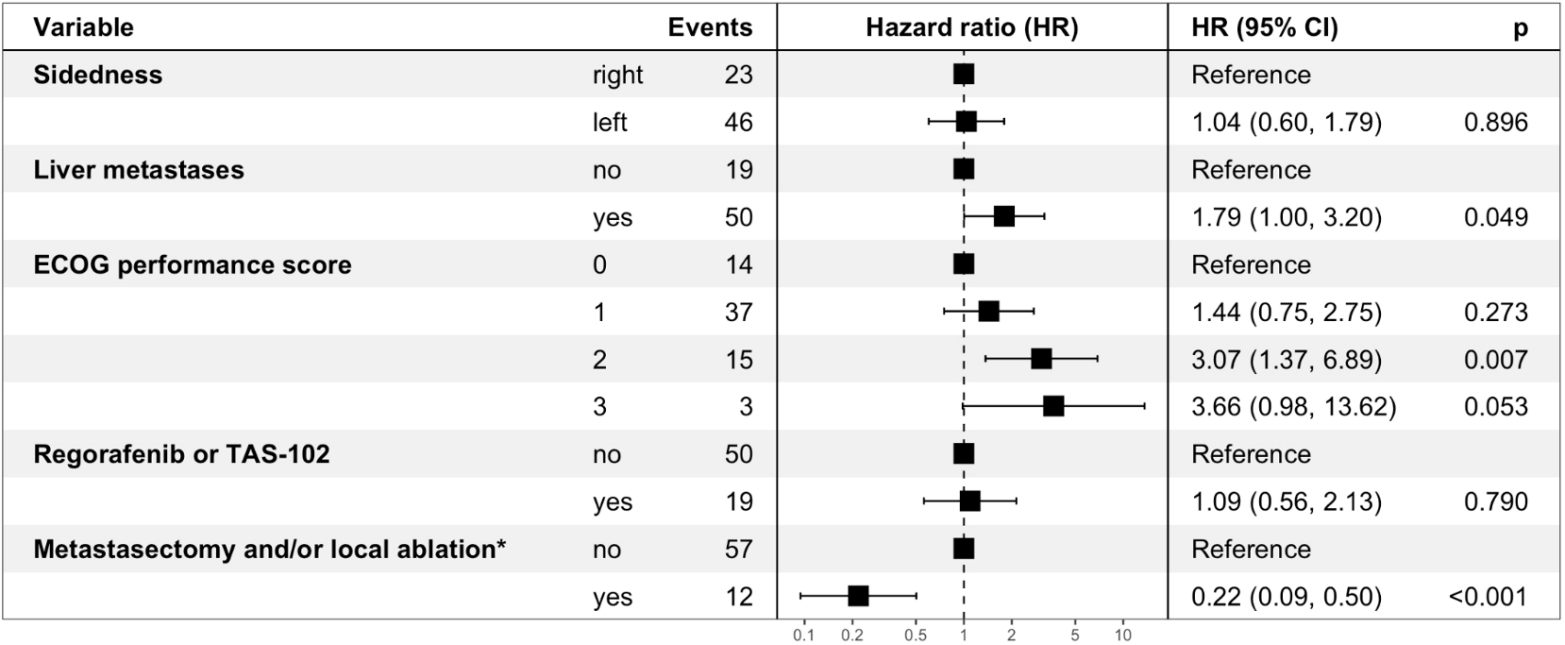
Multivariable analysis for overall survival – Forest Plot. ECOG performance score: Eastern Cooperative Oncology Group performance score. Regorafenib and/or TAS-102 exposure as well as metastasectomy and/or local ablative treatment were taken into consideration as time-dependent covariates. *involved organs: liver, lung, peritoneum, other.

In multivariable analysis, metastasectomy and/or local ablative treatment (yes versus no, HR: 0.22, p<0.001), the ECOG performance score (2 versus 0, HR: 3.07, p=0.007; 3 versus 0, HR: 3.66, p=0.053) and the presence of liver metastases (yes versus no, HR: 1.79, p=0.049) remained statistically significantly and independently associated with survival (**Figure 3**).

## Discussion

As the aging population is highly represented among mCRC patients and due to the paucity of trial-based recommendations, therapeutic decision making in elderly mCRC patients remains challenging in clinical practice. In our unicentric, retrospective analysis we characterized patient and tumor characteristics and investigated clinical outcome in a representative elderly patient cohort undergoing systemic therapy for mCRC. The distribution of age categories within our elderly mCRC cohort was comparable to records of the Tumor Registry of the Province of Salzburg between 2013 and 2020: 70-74 years: 31% versus 32%; 75-79 years: 36% versus 35%; 80-84 years: 27% versus 33%, ≥85 years: 6% versus 0%. It is noteworthy, that only 59% of mCRC patients ≥70 years of age received palliative systemic therapy in the Province of Salzburg.

Based on the findings of our unicentric analysis we provide further evidence that OS of elderly mCRC patients undergoing systemic therapy in the real-world setting (mOS of 25.6 months) is comparable to landmark clinical trials (7, 12) (mOS of 19 to 21 months, **Table A.4**). Metastasectomy and/or local ablative treatment with curative intent proved feasible in selected elderly patients and resulted in a significant and clinically meaningful OS benefit (HR: 0.22, p<0.001, **Figure 3**). Furthermore, the observed trend towards superior OS with an anti-EGFR-based therapy in left-sided mCRC when compared to anti-VEGF-based therapy or chemotherapy alone (**Figure 2D**) sheds further light on the predictive value of sidedness and corroborates the preference of anti-EGFR-based therapy also in elderly patients with RAS/BRAF wild-type left-sided disease.

In a cross-trial comparison between our retrospective analysis and a pooled analysis (13) of the TRIBE (14) and TRIBE2 (15) study, fewer patients received subsequent therapy lines in our elderly mCRC cohort (2L: 77% versus 52%, 3L: 53% versus 27%, 4L: 27% versus 12%, 5L: 11% versus 3%, **Figure 1**). It is noteworthy that the median age at mCRC diagnosis in the aforementioned studies (TRIBE: 60.0 and 60.5 years; TRIBE2: 60.0 and 61.0 years) was considerably lower when compared to our cohort (78 years, range: 70-90 years). However, the probability to receive subsequent systemic therapy was higher in our cohort compared to mCRC patients in the AVEX trial (7) (52% versus 37%).

The chemotherapy backbone in first line (doublet or triplet versus mono) did neither impact the number of subsequent therapy lines (p=0.697, **Figure 1**), nor had an impact on clinical outcome in the entire cohort (HR: 0.83, p=0.50) or among patients not eligible for metastasectomy and/or local ablative treatment (HR: 1.11, p=0.73). The latter findings are in line with the MRC FOCUS2 (16) and FFCD 2001-02 (17) trials, where the addition of oxaliplatin (16) or irinotecan (17) to 5-FU or capecitabine did not improve OS in elderly and/or frail mCRC patients, but significantly increased the frequency of grade 3-4 toxicities (17). In this regard, it is noteworthy that the ECOG PS in our elderly cohort was comparable to the study population of the MRC FOCUS2 trial (16): ECOG 0: 21%/21%, ECOG 1: 48%/50%, ECOG 2: 26%/29%, ECOG 3: 5%/0%.

Higher treatment-related toxicity rates with a doublet chemotherapy backbone and a higher frequency of comorbidities have also been observed with increasing age in the CALGB 80405 study (18). Age demonstrated as a considerable prognostic factor in the FIRE-3 study (19) (≥65 years: 25.9 versus <65 years: 29.3 months, p=0.02) and CALGB 80405 study (18) (≥70 years versus <70 years: HR 1.32, p<0.001). Within our study population (range: 70-90 years), older patients showed a trend towards a worse ECOG PS (p=0.087), however, age as a continuous parameter did not show any additional prognostic value among mCRC patients ≥70 years (**Table 2**).

A worse ECOG PS at mCRC diagnosis showed a statistically significant and independent association with inferior OS (2 versus 0, HR: 3.07; 3 versus 0: HR; 3.66; **Figure 3**). While classification into the ECOG PS categories (from 0: fully active to 4: completely disabled) can be rapidly performed in daily clinical practice in younger patients, the latter performance score assessment can be challenging in elderly cancer patients due to physicians’ varying conception of the usual performance spectrum of elderly people. Considerable disparities between patient-reported and physician-reported ECOG PS ratings exist (20) and there is also a poor agreement in ECOG PS ratings between clinicians (21). Other scores such as the Charlson Comorbidity Index (22), which includes age and multiple comorbidities and classifies into four risk categories, proved as predictors of survival in (m)CRC (23–25). However, our findings confirm the ECOG PS as a time-saving prognosticator and helpful tool for therapeutic decision-making (e.g. chemotherapy intensity) in daily clinical practice in elderly mCRC patients. The International Society of Geriatric Oncology recommends geriatric assessment in older cancer patients aiming at influencing treatment choice, predicting treatment-related complications and predicting clinical outcome. Geriatric assessment should include functional status, comorbidities, cognition, mental health status, fatigue, social status and support, nutrition, and the presence of geriatric syndromes (26). Based on the retrospective nature of our analysis, only the functional status was extracted from medical records and geriatric assessment was not feasible.

Contrary to the literature (27), sidedness was not prognostic among elderly mCRC patients in our cohort (left-sided versus right-sided, HR: 1.10 p=0.71, **Figure 2A**), which may be explained by the application of front-line anti-VEGF-based therapy in the majority of cases with right-sided (58%) as well as left-sided (51%) primary tumor localization. Furthermore, a higher percentage of patients with right-sided primary tumors underwent metastasectomy and/or local ablative treatment (right-sided: 33% versus left-sided: 15%, **Table A.3**). This stands in contrast to the secondary metastasectomy rate among patients ≥65 years in the FIRE-3 study (19) (right-sided: 8%-13% versus left-sided: 15%-26%).

A *post-hoc* analysis of the FIRE-3 study in the subgroup of patients ≥ 65 years (n=199) could neither corroborate the survival benefit of cetuximab versus bevacizumab in left-sided mCRC (33.2 months versus 27.5 months, HR: 0.86, p=0.38), nor the disadvantage of first-line cetuximab-based therapy in right-sided disease (16.6 months versus 23.6 months, HR: 1.1, p=0.87) (19). Liver surgery for colorectal metastases with curative intent in elderly mCRC patients can yield a comparable OS benefit as in the young population (28, 29). For elderly mCRC patients undergoing CRC liver metastases resection an incidence of 60- to 90-day mortality ranging between 4% and 8% has been reported in population-based studies (29, 30).

According to the RAXO study, a nationwide Finnish prospective intervention study, up to 41% of mCRC patients can be classified as resectable with curative intent either upfront or after conversion therapy irrespective of chronological age (31). In our cohort, metastasectomy and/or local ablative treatment were performed in 22% of patients with technically resectable disease extent and adequate performance status and yielded a clinically meaningful and independent OS benefit (HR: 0.22, **Figure 3**). This is in line with the secondary metastasectomy rate (18%) and the OS advantage (HR: 0.44) of elderly patients in the FIRE-3 study (19). The latter findings should encourage us to identify eligible patients for metastasectomy and/or local ablative treatment with curative intent in the elderly mCRC population. The presence of liver metastases was a significant and independent negative prognostic factor (HR: 1.79, p=0.05) – presumably mainly driven by non-resectable and non-liver-limited disease.

However, we would like to emphasize that in the FIRE-3 and CALGB 80405 studies elderly patients were defined by ≥65 years and ≥70 years, respectively, and were all deemed fit for a doublet chemotherapy backbone (18, 19). Data from the Cardiovascular Health Study corroborate an increasing prevalence of frailty with higher chronological age (32). Therefore, the FIRE-3 and CALGB 80405 mCRC populations may not properly reflect the elderly and often frail mCRC population in the real-world setting.

Within the inclusion period of our retrospective analysis (2009-2022), regorafenib (33) as well as TAS-102 (34) have been established as EMA- and FDA-approved third-line therapy options based on a survival benefit versus placebo, respectively. In our cohort, one out of five patients received regorafenib and/or TAS-102 during the course of disease (**Table 1**). Since the availability of regorafenib and TAS-102 within named patient programs or based on the respective EMA approval, our treatment strategy has not favored one drug over the other in the time interval between 2014 and 2022 (**Figure A.1**). However, based on the toxicity profile of regorafenib (33), an increased skeletal muscle loss (35) and a higher frequency of hospitalizations with regorafenib compared to TAS-102 (36), regorafenib should be used with caution in elderly mCRC patients. Treatment with regorafenib and/or TAS-102 did not result in a survival advantage when taken into account as a time-dependent covariate (yes versus no, HR: 1.09, p=0.79, **Figure 3**). According to the ESMO Magnitude of Clinical Benefit Scale (ESMO-MCBS) (37), which is based on the extent of OS gain, QoL and toxicities, TAS-102 (MCBS: 3) proved superior to regorafenib (MCBS: 1) (5).

The SUNLIGHT study, a randomized phase 3 study comparing TAS-102 versus TAS-102 in combination with bevacizumab for third-line treatment of refractory mCRC, has met its primary endpoint, demonstrating an OS benefit with TAS-102 plus bevacizumab (10.8 months versus 7.5 months, HR: 0.61, p<0.001) (38). Due to the acceptable safety profile of TAS-102 combined with bevacizumab in previous studies (9, 38, 39) this combination may become a new third-line standard in the near future, particularly suitable for the elderly and frail mCRC population.

The availability of further new treatment options (40,) (41) within the inclusion period (2009-2022) may have also contributed to the encouraging clinical outcome (mOS of 25.6 months) compared to the experimental arm of the AVEX trial (7) (mOS of 20.7 months, **Table A.4**).

Potential limitations of our study include the retrospective nature and the length of the inclusion period (2009-2022). Within the latter time span, biomarker refinement for established therapies (42), numerous new therapies for all-comers (33, 34) and biomarker-defined targeted-therapies (40, 41) changed daily clinical practice resulting in heterogenous treatments strategies in our elderly mCRC cohort. As a consequence, the predictive biomarker status is incomplete in a relevant number of patients. Furthermore, the implementation of sidedness into first-line decision making took place after the Annual ASCO Meeting 2016 (27, 43, 44), therefore, sidedness as a predictive biomarker could only be applied in less than half of our elderly mCRC patients. It is noteworthy, that elderly patients undergoing only a best supportive care strategy were excluded from our analysis. Although the number of included patients in our analysis (n=117) was limited, the sample size was comparable to the experimental arms of the AVEX (n=140) and PANDA (n=93) landmark trials (**Table A.4**).

## Conclusions

Clinical outcome among real-world elderly (≥70 years) mCRC patients is comparable to the results of first-line elderly mCRC landmark trials. First-line monochemotherapy plus targeted therapy based on sidedness and molecular status should be the treatment of choice. Based on proper patient selection, one out of five elderly mCRC patients qualifies for metastasectomy and/or local ablative treatment with curative intent. A doublet chemotherapy backbone +/- targeted therapy may be expedient in elderly mCRC patients who are candidates for metastasectomy and/or local ablative treatment. The latter ablative measures are feasible and yield a clinically meaningful survival benefit in selected elderly mCRC patients.

## Data availability statement

The raw data supporting the conclusions of this article will be made available by the authors on reasonable request.

## Ethics statement

The studies involving human participants were reviewed and approved by the Ethics Committee of the provincial government of Salzburg, Austria (415-E/2343/5-2018). Written informed consent for participation was not required for this study in accordance with the national legislation and the institutional requirements.

## Author contributions

FH: Conceptualization, Formal analysis, Investigation, Methodology, Project administration, Writing – original draft, Writing – review and editing. CD: Investigation, Writing – review and editing. GR: Formal analysis, Investigation, Methodology, Writing – review and editing. KS: Investigation, Writing – review and editing. RH: Investigation, Writing – review and editing. KE: Investigation, Writing – review and editing. DN: Investigation, Writing – review and editing. EK: Investigation, Writing – review and editing. MD: Investigation, Writing – review and editing. FR: Investigation, Writing – review and editing. RG: Funding Acquisition, Resources, Supervision, Writing – review and editing. LW: Conceptualization, Formal analysis, Funding acquisition, Investigation, Methodology, Project administration, Supervision, Writing – original draft, Writing – review and editing. All authors contributed to the article and approved the submitted version.

## Supplementary material

Supplementary Table 1 Association between 1L chemotherapy backbone and ECOG PS as well as age at mCRC diagnosis.

Supplementary Table 2 Elderly mCRC patients undergoing metastasectomy and/or local ablative treatment with curative intent (N=26) MWA: microwave ablation, RFA: radiofrequency ablation, SBRT: stereotactic body radiation therapy, TACE: transarterial chemoembolization.

Supplementary Table 3 Comparison of baseline characteristics between elderly mCRC patients undergoing metastasectomy and/or local ablative treatment versus not ^#^Mann-Whitney-U-Test. ECOG, Eastern Cooperative Oncology Group, mCRC, metastatic colorectal cancer.

Supplementary Table 4 Comparison of baseline characteristics and clinical outcome between the Salzburg elderly mCRC real-world cohort and elderly mCRC landmark trials.

Supplementary Figure 1 Regorafenib and TAS-102 exposure among elderly mCRC patients between 2014 and 2022 Cumulative cases of regorafenib (blue), TAS-102 (green) and total regorafenib and TAS-102 applications (red) between 2014 and 2022 among elderly mCRC patients.
