# Supplementary material for: Management of metastatic colorectal cancer in patients ≥70 years - a single center experience
Source: Front Oncol. 2023 Jul 25;13:1222951. doi: 10.3389/fonc.2023.1222951 (PMC10407548; doi:10.3389/fonc.2023.1222951)
Supplement: Supplementary Table 1 — Association between 1L chemotherapy backbone and ECOG PS as well as age at mCRC diagnosis. [file Table_1.docx]

**Table A.1 Association between 1L chemotherapy backbone and ECOG PS as well as age at mCRC diagnosis**

|  | **ECOG performance score** | | | | | |
| --- | --- | --- | --- | --- | --- | --- |
| **Chemotherapy backbone** | **0**  **(%)** | **1**  **(%)** | **2**  **(%)** | **3**  **(%)** | **Total** | **P=0.007** |
| Mono | 3 (12) | 11 (21) | 14 (47) | 3 (60) | **31** |  |
| Doublet or Triplet | 21 (88) | 42 (79) | 16 (53) | 2 (40) | **81** |  |
| Total | **24** | **53** | **30** | **5** | **112** |  |
|  | **Age category at mCRC diagnosis** | | | | | |
| **Chemotherapy backbone** | **70-74**  **years** | **75-79 years** | **80-84 years** | **≥85 years** | **Total** | **P<0.001** |
| Mono | 2 (6) | 8 (19) | 15 (48) | 7 (100) | **32** |  |
| Doublet or Triplet | 34 (94) | 33 (81) | 16 (52) | 0 (0) | **83** |  |
| Total | **36** | **41** | **31** | **7** | **115** |  |
