# Supplementary material for: Management of metastatic colorectal cancer in patients ≥70 years - a single center experience
Source: Front Oncol. 2023 Jul 25;13:1222951. doi: 10.3389/fonc.2023.1222951 (PMC10407548; doi:10.3389/fonc.2023.1222951)
Supplement: Supplementary Table 2 — Elderly mCRC patients undergoing metastasectomy and/or local ablative treatment with curative intent (N=26) MWA: microwave ablation, RFA: radiofrequency ablation, SBRT: stereotactic body radiation therapy, TACE: transarterial chemoembolization. [file Table_2.docx]

**Table A.2 Elderly mCRC patients undergoing metastasectomy and/or local ablative treatment with curative intent (N=26)**

| **Procedure** | **Number of patients** | **Involved organ** | | | |
| --- | --- | --- | --- | --- | --- |
|  |  | **Liver** | **Lung** | **Peritoneum** | **other** |
| **Metastasectomy** |  |  |  |  |  |
| 1^st^ procedure | 23 | 15 | 2 | 2 | 4 |
| 2^nd^ procedure | 9 | 3 | 4 | 1 | 1 |
| 3^rd^ procedure | 2 | - | 1 | - | 1 |
| **RFA/MWA** |  |  |  |  |  |
| 1 procedure | 6 | 6 | - | - | - |
| **SBRT** |  |  |  |  |  |
| 1 procedure | 6 | 3 | 3 | - | - |
| **TACE** |  |  |  |  |  |
| 1 procedure | 2 | 2 | - | - | - |

MWA: microwave ablation, RFA: radiofrequency ablation, SBRT: stereotactic body radiation therapy, TACE: transarterial chemoembolization
