# Supplementary material for: Management of metastatic colorectal cancer in patients ≥70 years - a single center experience
Source: Front Oncol. 2023 Jul 25;13:1222951. doi: 10.3389/fonc.2023.1222951 (PMC10407548; doi:10.3389/fonc.2023.1222951)
Supplement: Supplementary Table 3 — Comparison of baseline characteristics between elderly mCRC patients undergoing metastasectomy and/or local ablative treatment versus not #Mann-Whitney-U-Test. ECOG, Eastern Cooperative Oncology Group, mCRC, metastatic colorectal cancer. [file Table_3.docx]

**Table A.3 Comparison of baseline characteristics between elderly mCRC patients undergoing metastasectomy and/or local ablative treatment versus not**

| **Parameter** | **No local ablation therapy**  **N=91 (%)** | **Local ablation therapy**  **N=26 (%)** | **P-value** |
| --- | --- | --- | --- |
| Age (median)  Range | 78  70-90 | 76.5  70-82 | 0.157^#^ |
| Sex  Female  Male | 37 (41)  54 (59) | 11 (42)  15 (58) | 0.880 |
| ECOG at mCRC diagnosis  0  1  2  3  NA | 18 (20)  41 (46)  25 (28)  5 (6)  2 | 6 (24)  14 (56)  5 (20)  0 (0)  1 | 0.490 |
| Sidedness  Left  Right | 61 (67)  30 (33) | 11 (42)  15 (58) | 0.379 |
| Time point of metastases detection  Synchronous  Metachronous | 71 (78)  20 (22) | 16 (62)  10 (38) | 0.090 |
| KRAS status  Wild-type  Mutant  NA | 36 (46)  42 (54)  13 | 13 (50)  13 (50)  0 | 0.734 |
| Liver metastases  Yes  No | 63 (69)  28 (31) | 17 (65)  9 (35) | 0.710 |
| Lung metastases  Yes  No | 34 (37)  57 (63) | 6 (23)  20 (77) | 0.176 |
| Peritoneal metastases  Yes  No | 19 (21)  72 (79) | 3 (11)  23 (89) | 0.282 |
| 1L chemotherapy backbone  Mono chemotherapy  Doublet or triplet chemotherapy  NA (anti-PD-1 therapy) | 29 (33)  60 (67)  2 | 3 (11)  23 (89)  0 | 0.035 |
| 1L anti-VEGF or anti-EGFR therapy  None  Anti-VEGF  Anti-EGFR | 28 (31)  48 (53)  15 (16) | 7 (27)  13 (50)  6 (23) | 0.734 |

^#^Mann-Whitney-U-Test

ECOG: Eastern Cooperative Oncology Group, mCRC: metastatic colorectal cancer
