# Supplementary material for: Management of metastatic colorectal cancer in patients ≥70 years - a single center experience
Source: Front Oncol. 2023 Jul 25;13:1222951. doi: 10.3389/fonc.2023.1222951 (PMC10407548; doi:10.3389/fonc.2023.1222951)
Supplement: Supplementary Table 4 — Comparison of baseline characteristics and clinical outcome between the Salzburg elderly mCRC real-world cohort and elderly mCRC landmark trials. [file Table_4.docx]

**Table A.4 Comparison of baseline characteristics and clinical outcome between the Salzburg elderly mCRC real-world cohort and elderly mCRC landmark trials**

|  | **Salzburg elderly mCRC cohort** | **AVEX trial** | **SOLSTICE trial** | **PANDA trial** |
| --- | --- | --- | --- | --- |
| **Study design** | Retrospective, unicentric | Open-label, multicentric, randomized phase III | Open-label, multicentric, randomized phase III | Open-label, multicentric, randomized phase II |
| **1L-treatment arm** | Mono or doublet or triplet chemotherapy  +/- targeted therapy | Capecitabin + bevacizumab | Capecitabin + bevacizumab | FU/LV + panitumumab |
| **Inclusion criterion** | ≥70 years | ≥70 years | ≥70 years  and/or  frail patients  and/or  low tumor burden | ≥70 years |
| **Number of patients** | N=117 | N=140 | N=430  (N=259  ≥70 years) | N=93 |
| **Median age** | 78 | 76 | 73 | 77 |
| **ECOG PS**  **0**  **1**  **2**  **3** | 21%  48%  26%  5% | 51%  42%  7%  0% | 23%  58%  19%  <1% | NA |
| **Sidedness**  **Left-sided**  **Right-sided** | 65%  35% | NA  NA | 70%  30% | 79%  21% |
| **Liver metastases at mCRC diagnosis** | 68% | 63% | 65% | NA |
| **mOS (months)**  **(95% CI)** | 25.6  (21.8-29.4) | 20.7  (17.0-26.0) | NA | NA |
