# Supplementary figures and images for: Management of metastatic colorectal cancer in patients ≥70 years - a single center experience
Source: Front Oncol. 2023 Jul 25;13:1222951. doi: 10.3389/fonc.2023.1222951 (PMC10407548; doi:10.3389/fonc.2023.1222951)

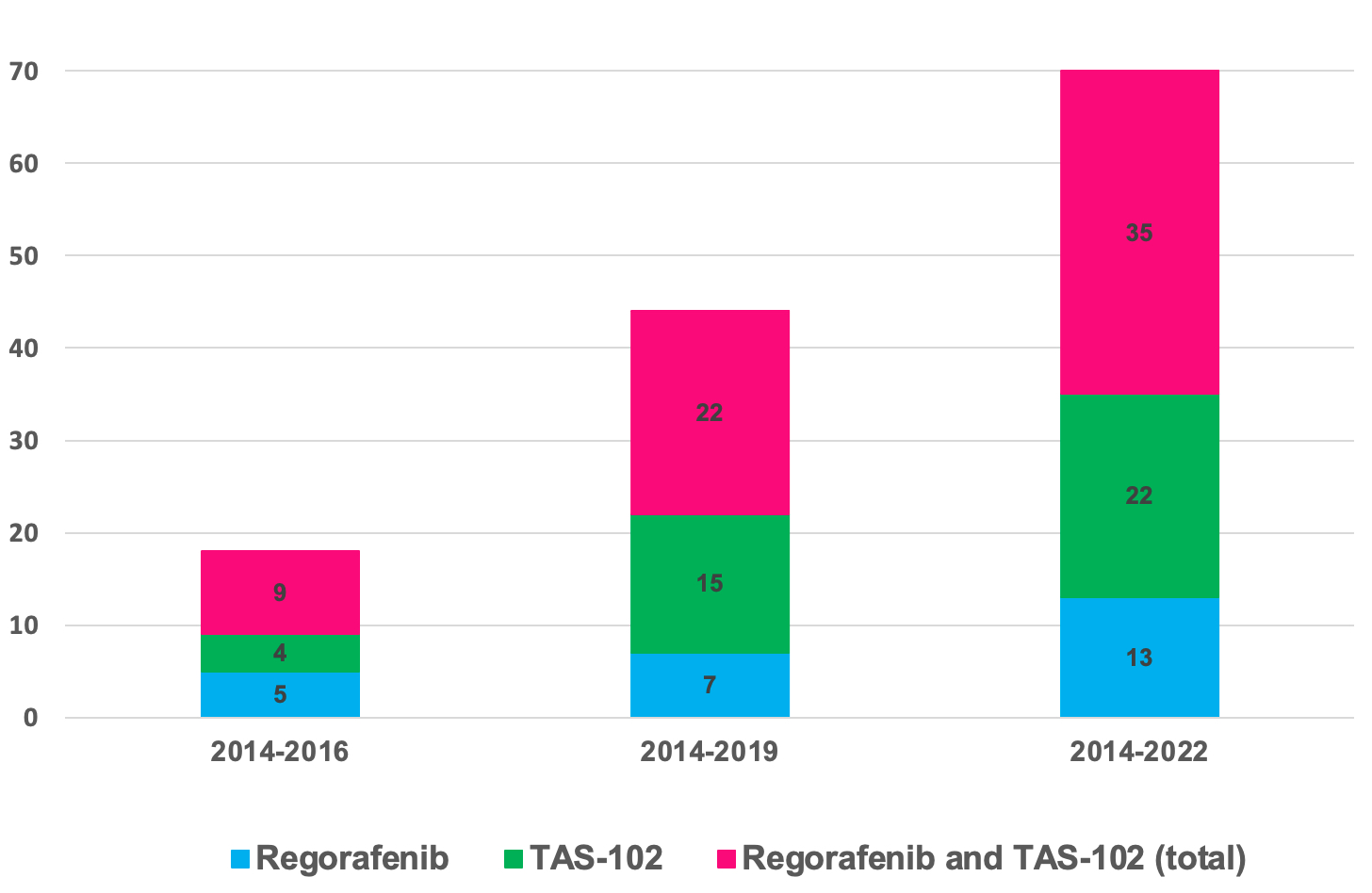

Supplement: Supplementary Figure 1 — Regorafenib and TAS-102 exposure among elderly mCRC patients between 2014 and 2022 Cumulative cases of regorafenib (blue), TAS-102 (green) and total regorafenib and TAS-102 applications (red) between 2014 and 2022 among elderly mCRC patients. [file Image_1.jpeg]
